# Molecular pathogenicity of 1-nonadecene and l-lactic acid, unique metabolites in radicular cysts and periapical granulomas

**DOI:** 10.1038/s41598-023-37945-w

**Published:** 2023-07-03

**Authors:** Alaa M. Altaie, Mohammad G. Mohammad, Mohamed I. Madkour, Mohammed Amjed AlSaegh, Manju Nidagodu Jayakumar, Aghila Rani K.G., A. R. Samsudin, Rabih Halwani, Rifat A. Hamoudi, Sameh S. M. Soliman

**Affiliations:** 1 Research Institute for Medical and Health Sciences, University of Sharjah, P.O. Box 27272, Sharjah, United Arab Emirates; 2 Department of Clinical Sciences, College of Medicine, University of Sharjah, P.O. Box 27272, Sharjah, United Arab Emirates; 3 Department of Medical Laboratory Sciences, College of Health Sciences, University of Sharjah, P.O. Box 27272, Sharjah, United Arab Emirates; 4 Department of Oral and Craniofacial Health Sciences, College of Dental Medicine, University of Sharjah, P.O. Box 27272, Sharjah, United Arab Emirates; 5 Division of Surgery and Interventional Science, University College London, London, United Kingdom; 6 ASPIRE Precision Medicine Research Institute Abu Dhabi, University of Sharjah, Sharjah, United Arab Emirates; 7 Department of Medicinal Chemistry, College of Pharmacy, University of Sharjah, P.O. Box 27272, Sharjah, United Arab Emirates

**Keywords:** Cell biology, Immunology, Molecular biology, Medical research

## Abstract

Recently, 1-nonadecene and l-lactic acid were identified as unique metabolites in radicular cysts and periapical granuloma, respectively. However, the biological roles of these metabolites were unknown. Therefore, we aimed to investigate the inflammatory and mesenchymal-epithelial transition (MET) effects of 1-nonadecene, and the inflammatory and collagen precipitation effects of l-lactic acid on both periodontal ligament fibroblasts (PdLFs) and peripheral blood mononuclear cells (PBMCs). PdLFs and PBMCs were treated with 1-nonadecene and l-lactic acid. Cytokines’ expression was measured using quantitative real-time polymerase chain reaction (qRT-PCR). E-cadherin, N-cadherin, and macrophage polarization markers were measured using flow cytometry. The collagen, matrix metalloproteinase (MMP)-1, and released cytokines were measured using collagen assay, western blot, and Luminex assay, respectively. In PdLFs, 1-nonadecene enhances inflammation through the upregulation of some inflammatory cytokines including IL-1β, IL-6, IL-12A, monocyte chemoattractant protein (MCP)-1, and platelet-derived growth factor (PDGF) α. 1-Nonadecene also induced MET through the upregulation of E-cadherin and the downregulation of N-cadherin in PdLFs. 1-Nonadecene polarized macrophages to a pro-inflammatory phenotype and suppressed their cytokines’ release. l-lactic acid exerted a differential impact on the inflammation and proliferation markers. Intriguingly, l-lactic acid induced fibrosis-like effects by enhancing collagen synthesis, while inhibiting MMP-1 release in PdLFs. These results provide a deeper understanding of 1-nonadecene and l-lactic acid’s roles in modulating the microenvironment of the periapical area. Consequently, further clinical investigation can be employed for target therapy.

## Introduction

Microenvironment (vasculature, myoepithelial cells, fibroblasts, extracellular matrix, and immune cells) of a lesion plays important roles in its initiation and pathogenicity^1^. Metabolism and metabolites represent a major contributor in modulation of such microenvironment^2^. Recently, we have identified 1-nonadecene and l-lactic acid as the highest unique metabolites in radicular cysts and periapical granulomas, respectively^3^. The contribution of l-lactic acid in the formation of facial granulomatous tissue after poly-l-lactic acid injection was previously reported^4^. However, its mechanism of pathogenesis or contribution in other tissues have never been tested. On the other hand, 1-nonadecene was biologically identified for the first time in radicular cysts^3^. 1-Nonadecene is a natural metabolite identified in bacteria^5^ and fungi^6^. There are, however, insufficient data available on its biological and immunological activities in human cells. Therefore, we aimed to investigate the immunological and metabolic effects of these two metabolites on both PdLFs and macrophages. PdLFs represent the most predominant cells within the periapical area that play a critical role in the remodeling process and wound healing^7^. Furthermore, the periapical area was predominantly infiltrated with lymphocytes, plasma cells, and macrophages^8^.

The inner layer of radicular cyst cavity is composed of stratified squamous epithelium with an outer wall of dense fibrous capsule infiltrated with chronic inflammatory cells^9^. These epithelial cells are expected to arise from the epithelial rests of Malassez^10^, while a recent study reported the possible conversion of dermal fibroblasts to epithelial cells^11^. However, the underlying mechanism is not reported. On the other hand, periapical granulomas consist of an organized profuse collagen fibers in diverse directions appearing as irregular dense connective tissue with vascular elements^12^.

PdLFs can act as immune cells and secret different cytokines^13^. The main reparative function of PdLFs is to secrete extracellular matrix components such as collagen, that builds up the periodontal ligament and its fibers^14^. Besides, macrophages are important components of the inflammatory process^15^ in response to microbial infiltration in the periapical area and hence shaping up a specific periapical lesion^16^. Microbial metabolites are more predominantly contributing to such inflammatory conditions^17^.

In response to microbial entrance to the apical area, the host mounts a series of immunological and metabolic reactions that result in the destruction of periapical tissue and the development of cysts and granulomas^3^. Radicular cysts and periapical granulomas are among the most frequently occurring pathological lesions in the alveolar bone, accounting for 95% of the periapical radiolucency^18^. Interestingly, a radicular cyst may evolve from a previous periapical granuloma^19^, indicating a metabolic change in the lesion microenvironments for the induction of each lesion^3^. Thus, understanding the inflammatory and immunological roles of the major unique metabolites in the initiation of such lesions may provide potential diagnostic and therapeutic values.

## Results

The effects of different concentrations of 1-nonadecene and l-lactic acid on PdLFs and PBMCs were investigated as follows:

### 1-Nonadecene revealed an immunological activation of PdLFs and PBMCs to express inflammatory cytokines

**Figure 1:**
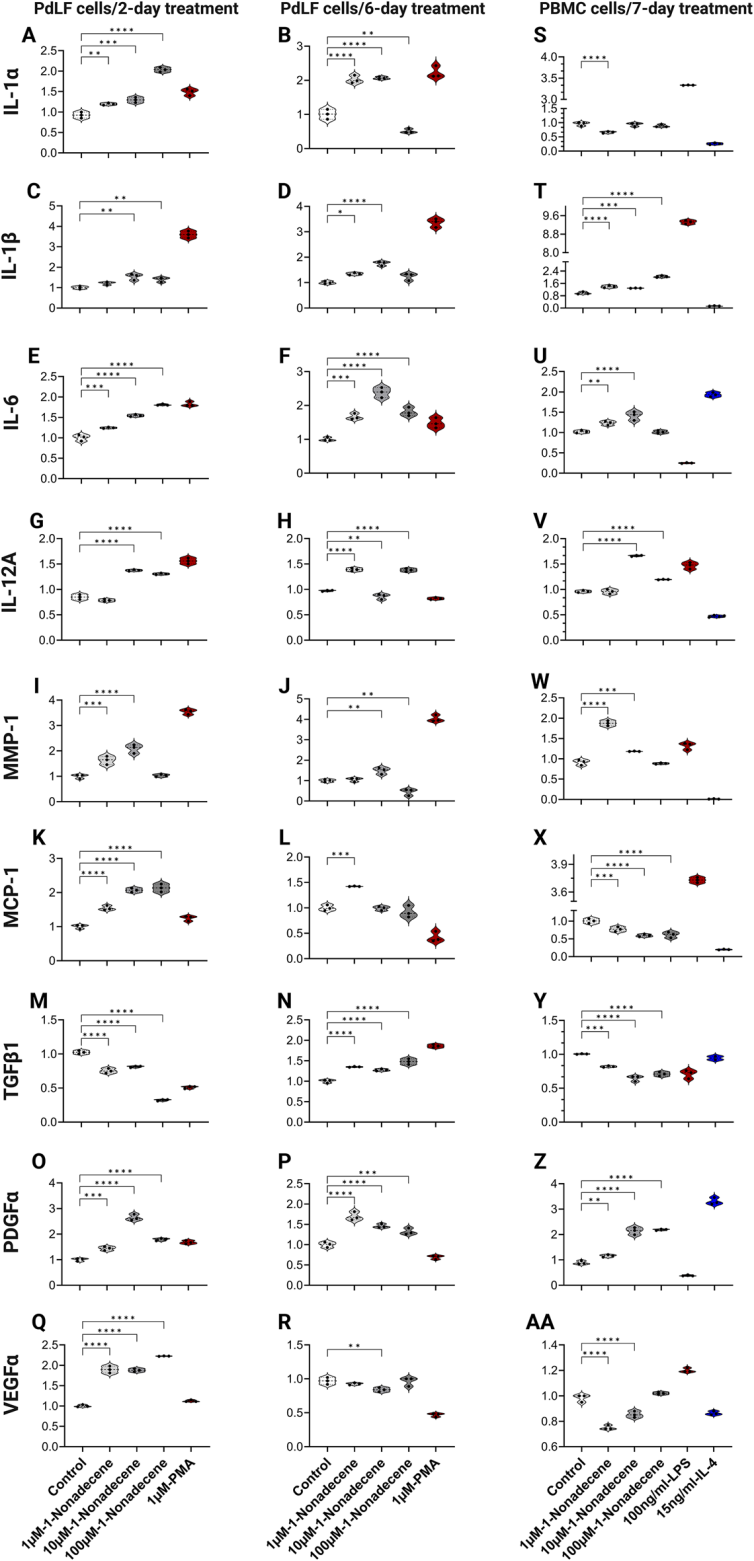
Inflammatory cytokines’ expression in PdLFs and PBMCs in response to 1-nonadecene treatment. (**A**,**C**,**E**,**G**,**I**,**K**,**M**,**O**,**Q**) are the gene expression of cytokines in PdLFs treated with 1 µM, 10 µM, 100 µM 1-nonadecene, and 1 µM PMA for 2 days. (**B**,**D**,**F**,**H**,**J**,**L**,**N**,**P**,**R**) are the gene expression of cytokines in PdLFs treated with 1 µM, 10 µM, 100 µM 1-nonadecene, and 1 µM PMA for 6 days. (**S**–**AA**) are the gene expression of cytokines in PBMCs treated with 1 µM, 10 µM, 100 µM 1-nonadecene, and 100 ng/ml LPS, and 15 ng/ml IL-4 for 7 days. All cells were treated in triplicate. The data were analyzed using one-way analysis of variance (ANOVA) and Dunnett's multiple comparisons test. *P*-value < 0.05 was considered significant.

The gene expression of IL-1α, IL-1β, IL-6, IL12A, MMP-1, MCP-1, transforming growth factor (TGF)β1, PDGFα, and vascular endothelial growth factor (VEGF) α were investigated in PdLFs and PBMCs following the treatment by 1-nonadecene. In PdLFs, 1-nonadecene caused significant upregulation at 1 µM and 10 µM for IL-1α after 2- and 6-day treatments (Fig. 1A,B), at 10 µM for IL-1β after 2- and 6-day treatments (Fig. 1C,D), and at all concentrations for IL-6 after 2- and 6-day treatments (Fig. 1E,F). IL-12A was significantly upregulated at 10 µM and 100 µM after 2-day treatment (Fig. 1G) and at 1 µM and 100 µM after 6-day treatment (Fig. 1H). For MMP-1, the upregulation was observed at 1 µM and 10 µM after 2-day treatment and at 10 µM after 6-day treatment (Fig. 1I,J). MCP-1 was significantly upregulated at all concentrations after 2-day treatment (Fig. 1K) and at 1 µM after 6-day treatment (Fig. 1L). Controversial significant upregulation and downregulation in TGFβ1 expression were observed after 2- and 6-day treatments, respectively (Fig. 1M,N). The main proliferation marker, PDGFα, was significantly upregulated at all concentrations after 2- and 6-day treatments (Fig. 1O,P). For VEGFα, a significant upregulation was found at all concentrations after 2-day treatment (Fig. 1Q), while a significant downregulation at 10 µM after 6-day treatment was observed (Fig. 1R). In PBMCs, although a significant downregulation of IL-1α at 1 µM 1-nonadecene was found (Fig. 1S), all concentrations of 1-nonadecene caused significant upregulation in the gene expression of IL-1β (Fig. 1T), and IL-6 at 1 µM and 10 µM (Fig. 1U). Significant upregulation was observed for IL-12A at 10 µM and 100 µM (Fig. 1V) and for MMP-1 at 1 µM and 10 µM (Fig. 1W). Conversely, MCP-1 and TGFβ1 were significantly downregulated at all concentrations (Fig. 1X,Y). While PDGFα was significantly upregulated (Fig. 1Z), VEGFα was significantly downregulated (Fig. 1AA).

### 1-Nonadecene induced MET in PdLFs

**Figure 2:**
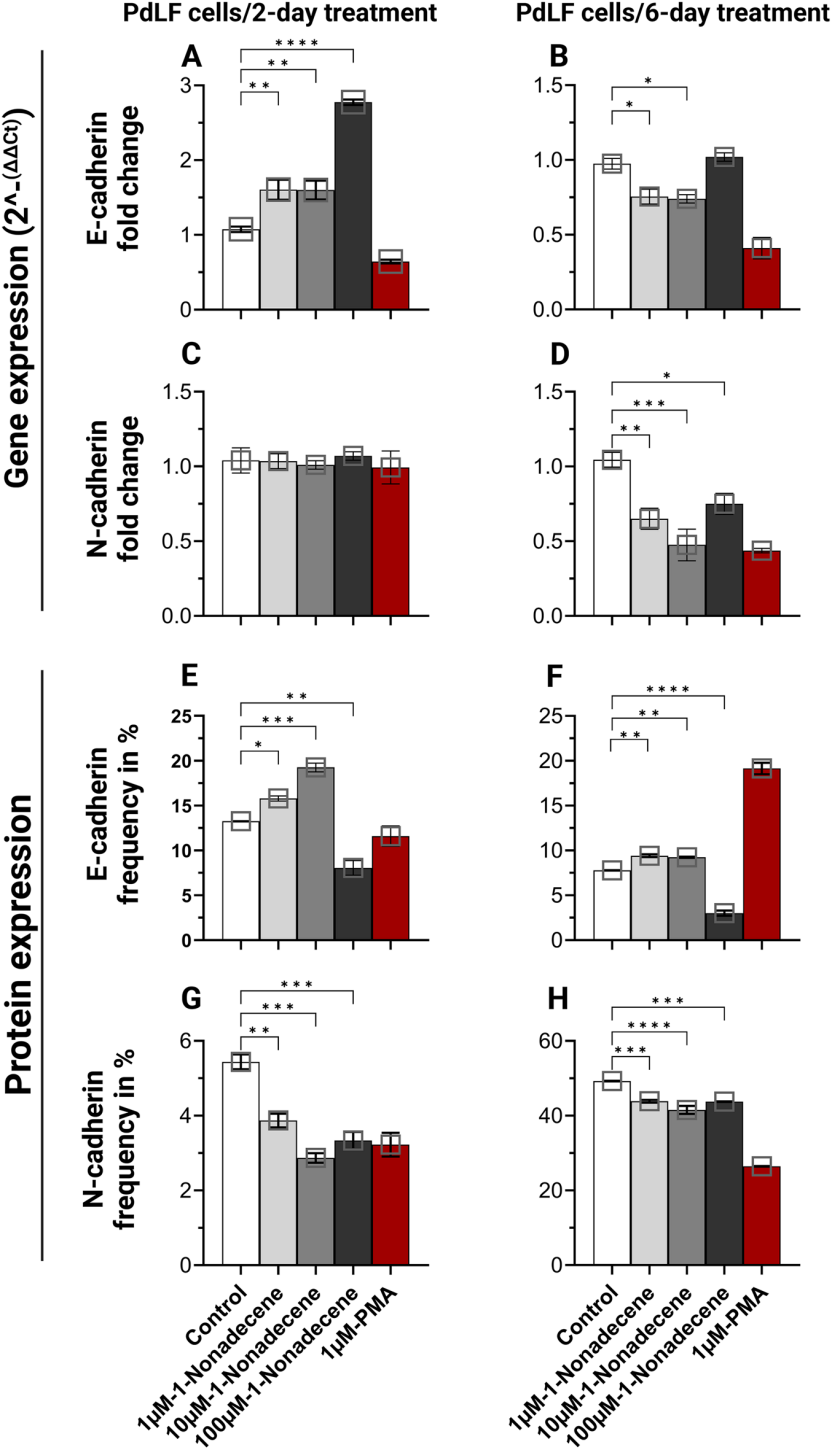
E-cadherin and N-cadherin gene and protein expression from PdLFs in response to 1-nonadecene treatment. PdLFs were treated with 1 µM, 10 µM, and 100 µM 1-nonadecene for 2 and 6 days as daily replacement of the metabolite. 1 µM phorbol 12-myristate 13-acetate (PMA) was used as positive and negative controls for N-cadherin and E-cadherin, respectively. The figure shows the fold change in the gene expression of PdLFs of (**A**) E-cadherin for 2 days, (**B**) E-cadherin for 6 days, (**C**) N-cadherin for 2 days, and (**D**) N-cadherin for 6 days. Frequency of E-cadherin and N-cadherin surface protein expression was showen using flow cytometry assay of (**E**) E-cadherin for 2 days, (**F**) E-cadherin for 6 days, (**G**) N-cadherin for 2 days, and (**H**) N-cadherin for 6 days. The cells were treated in triplicate. The data were analyzed using one-way ANOVA and Dunnett's multiple comparisons test. *P*-value < 0.05 was considered significant.

At the gene expression level, E-cadherin was significantly upregulated at all concentrations after only 2-day treatment (Fig. 2A and Supplementary Fig. 1), while downregulated at 1 µM and 10 µM after 6-day treatment (Fig. 2B and Supplementary Fig. 1). Conversely, N-cadherin did not show any change after 2-day treatment (Fig. 2C and Supplementary Fig. 1) but was significantly downregulated at all concentrations after 6-day treatment (Fig. 2D and Supplementary Fig. 1). The expression of E-cadherin was significantly increased at both 1 µM and 10 µM after 2- and 6-day treatments (Fig. 2E,F and Supplementary Fig. 1). On the other hand, significant decrease in N-cadherin surface protein was observed at all concentrations after 2- and 6-day treatments (Fig. 2G,H and Supplementary Fig. 1).

### 1-Nonadecene polarized PBMCs to pro-inflammatory/classical macrophage (M1)

To identify the proinflammatory response of PBMCs due to 1-nonadecene treatment, PBMCs were treated with 1 µM, 10 µM, and 100 µM 1-nonadecene for 7 days. 1 µM and 100 µM significantly polarized macrophages to classical phenotype (CD14^high^/CD16^low^) (*P*˂0.0001), while all concentrations significantly caused the upregulation of classical/HLA-DR/CD86, and classical/CD86 (*P*˂0.0001) (Supplementary Fig. 2A). Significant upregulation was found for classical/CD163 expression at 1 µM and 10 µM (*P*˂0.0001) and for classical/CD206 at 10 µM and 100 µM (Supplementary Fig. 2A). The anti-inflammatory/non-classical macrophage (M2) polarization (CD16^high^/CD14^low^) was not observed at all concentrations of 1-nonadecene but the activated form of non-classical/HLA-DR/CD86 at 1 µM and 10 µM (*P*˂0.0001) (Supplementary Fig. 2B) and non-classical/CD163 markers were significantly upregulated at all concentrations (Supplementary Fig. 2B). Non-classical/CD206 expression showed significant upregulation at 100 µM only (Supplementary Fig. 2B).

### The release of cytokines from PBMCs was correlated to 1-nonadecene’s effect

To validate whether 1-nonadecene can induce the pro-inflammatory cytokines from PBMCs. PBMCs were treated with 1 µM, 10 µM, and 100 µM 1-nonadecene for 7 days. As illustrated in Supplementary Fig. 3A and B, the concentrations of IL-1β and IL-6 did not change, while IL-10 was significantly reduced at all concentrations in the treated PBMCs (Supplementary Fig. 3C). Although IL-12p70 and IL-13 did not show significant change (Supplementary Fig. 3D, E), CXCL9 and CXCL10 were significantly reduced at all concentrations of 1-nonadecene (*P*˂0.0001) (Supplementary Fig. 3F,G). CCL17 did not show any change (Supplementary Fig. 3H), while CCL22 concentration was significantly reduced (*P*˂0.001) (Supplementary Fig. 3I). IL-23 and tumor necrosis factor (TNF)-α were also significantly reduced at 10 µM and 100 µM 1-nonadecene (Supplementary Fig. 3 J,K), while interferon (IFN)-γ did not change (Supplementary Fig. 3L).

### Cytokines expression in PdLFs and PBMCs was downregulated in response to l-lactic acid

**Figure 3:**
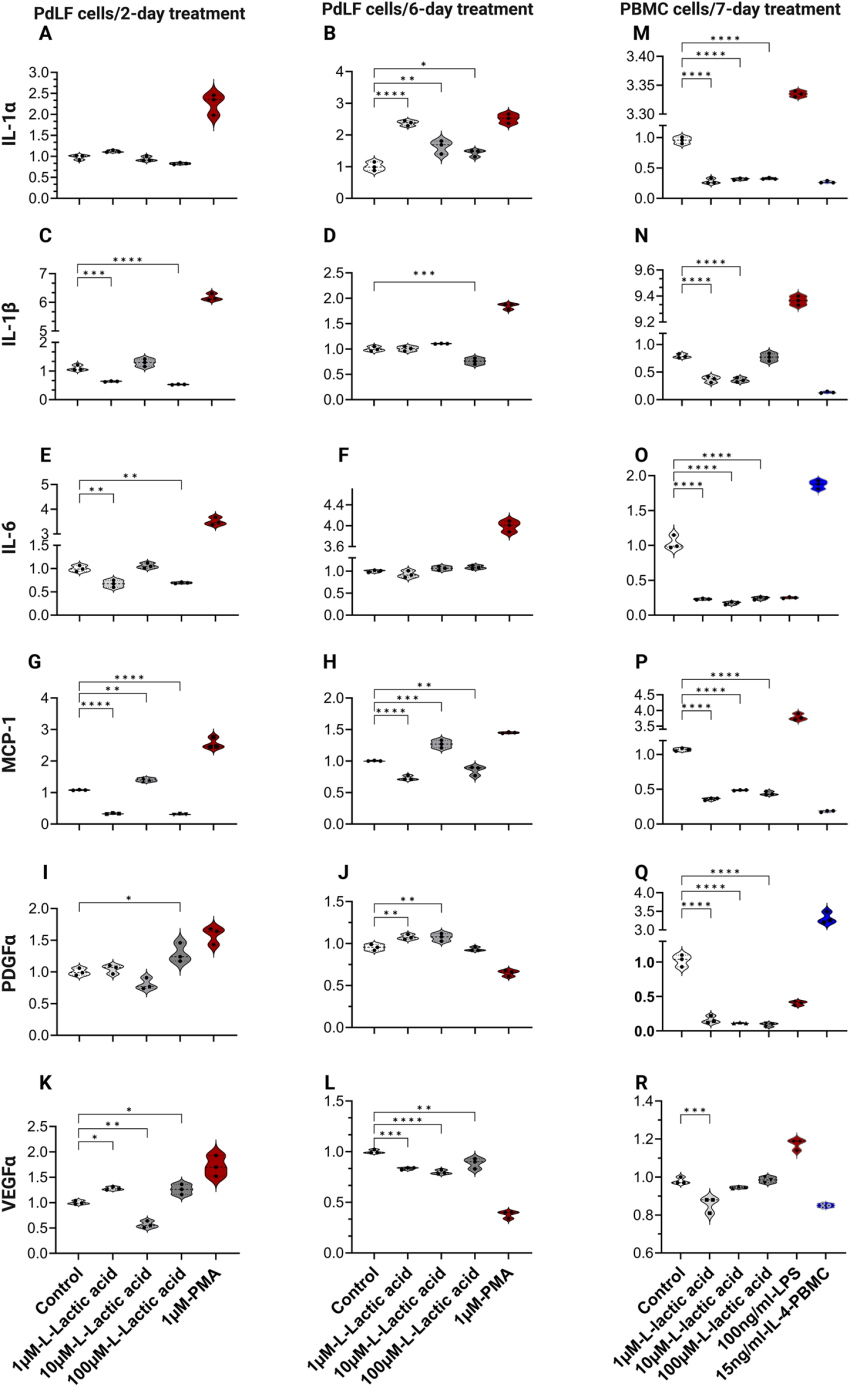
Inflammatory cytokines’ expression in PdLFs and PBMCs in response to L-lactic acid treatment. (**A**,**C**,**E**,**G**,**I**,**K**) are the gene expression of cytokines in PdLFs treated with 1 µM, 10 µM, 100 µM L-lactic acid, and 1 µM PMA for 2 days. (**B**,**D**,**F**,**H**,**J**,**L**) are the gene expression of cytokines in PdLFs treated with 1 µM, 10 µM, 100 µM L-lactic acid, and 1 µM PMA for 6 days. (**M–R**) are the gene expression of cytokines in PBMCs treated with 1 µM, 10 µM, 100 µM L-lactic acid, 100 ng/ml LPS, and 15 ng/ml IL-4 for 7 days. All cells were treated in triplicate. The data were analyzed using one-way ANOVA and Dunnett's multiple comparisons test. *P*-value < 0.05 was considered significant.

The gene expression of IL-1α, IL-1β, IL-6, MCP-1, PDGFα, and VEGFα were investigated in PdLFs and PBMCs following the treatment by l-lactic acid. In PdLFs, although IL-1α did not show significant change after 2-day treatment (Fig. 3A), significant upregulation at all concentrations were found after 6-day treatment (Fig. 3B). Significant downregulation in the gene expression was observed for IL-1β following 2- and 6-day treatments (Fig. 3C,D). For IL-6, the significant downregulation was observed following 2-day treatment (*P*˂0.01) (Fig. 3E), while following 6-day treatment, no significant change was observed (Fig. 3F). MCP-1 was downregulated at 1 µM and 100 µM, but significantly upregulated at 10 µM following 2- and 6-day treatments (Fig. 3G,H). For PDGFα, significant upregulation was found at some concentrations following 2- and 6-day treatments (Fig. 3I,J), while for VEGFα, a significant downregulation at 10 µM was found following 2-day treatment (Fig. 3K) and at all concentrations following 6-day treatment (Fig. 3L). In PBMCs, prominent downregulation in the gene expression of IL-1α, IL-1β, IL-6, MCP-1, PDGFα, and VEGFα was also observed (Fig. 3M–R).

### l-lactic acid simultaneously upregulated collagen precipitation and reduced its degradation through MMP-1 downregulation in PdLFs

**Figure 4:**
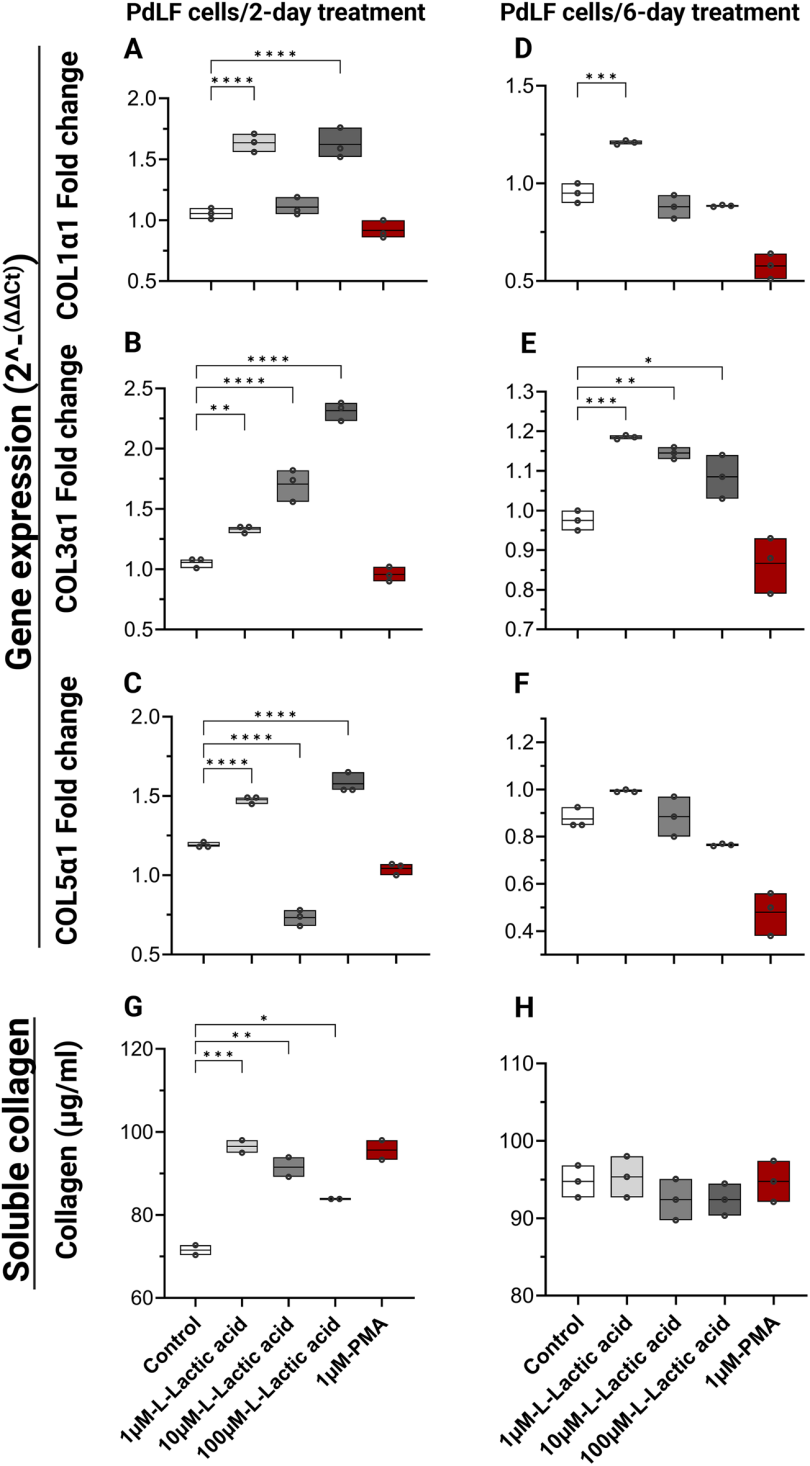
Collagen gene-expression and production from PdLFs in response to L-lactic acid treatment. PdLFs were treated with 1 µM, 10 µM, and 100 µM L-lactic acid for 2 and 6 days. 1 µM phorbol 12-myristate 13-acetate (PMA) was used as a positive control for collagen expression and production. The figure shows the fold change in gene expression in PdLFs for 2 days of (**A**) Collagen (COL)1α1, (**B**) COL3α1, and (**C**) COL5α1. Fold change in gene expression in PdLFs for 6 days of (**D**) COL1α1, (**E**) COL3α1, and (**F**) COL5α1. Soluble collagen concentration in µg/ml in PdLFs (**G**) for 2 days, and (**H**) for 6 days. The data were analyzed using one-way ANOVA and Dunnett's multiple comparisons test. The cells were treated in triplicate *P*-value < 0.05 was considered significant.

l-lactic acid at 1 µM and 100 µM caused significant upregulation in the expression of gene coding collagen (COL1α1, COL3α1, and COL5α1) after 2-day treatment, while 10 µM caused similar upregulation of only COL3α1 (Fig. 4A–C). While 1 µM l-lactic acid caused significant upregulation of COL1α1, all other concentrations caused upregulation of COL3α1 gene expression following 6-day treatment (Fig. 4D,E). However, following 6-day treatment, no significant change was observed for COL5α1 gene expression (Fig. 4F). Soluble collagen production in PdLFs was also significantly increased after 2-day treatment with 1 µM (*P* = 0.0005), 10 µM (*P* = 0.001), and 100 µM (*P* = 0.01) (Fig. 4G), while after 6 days, there was no significant change (Fig. 4H).

**Figure 5:**
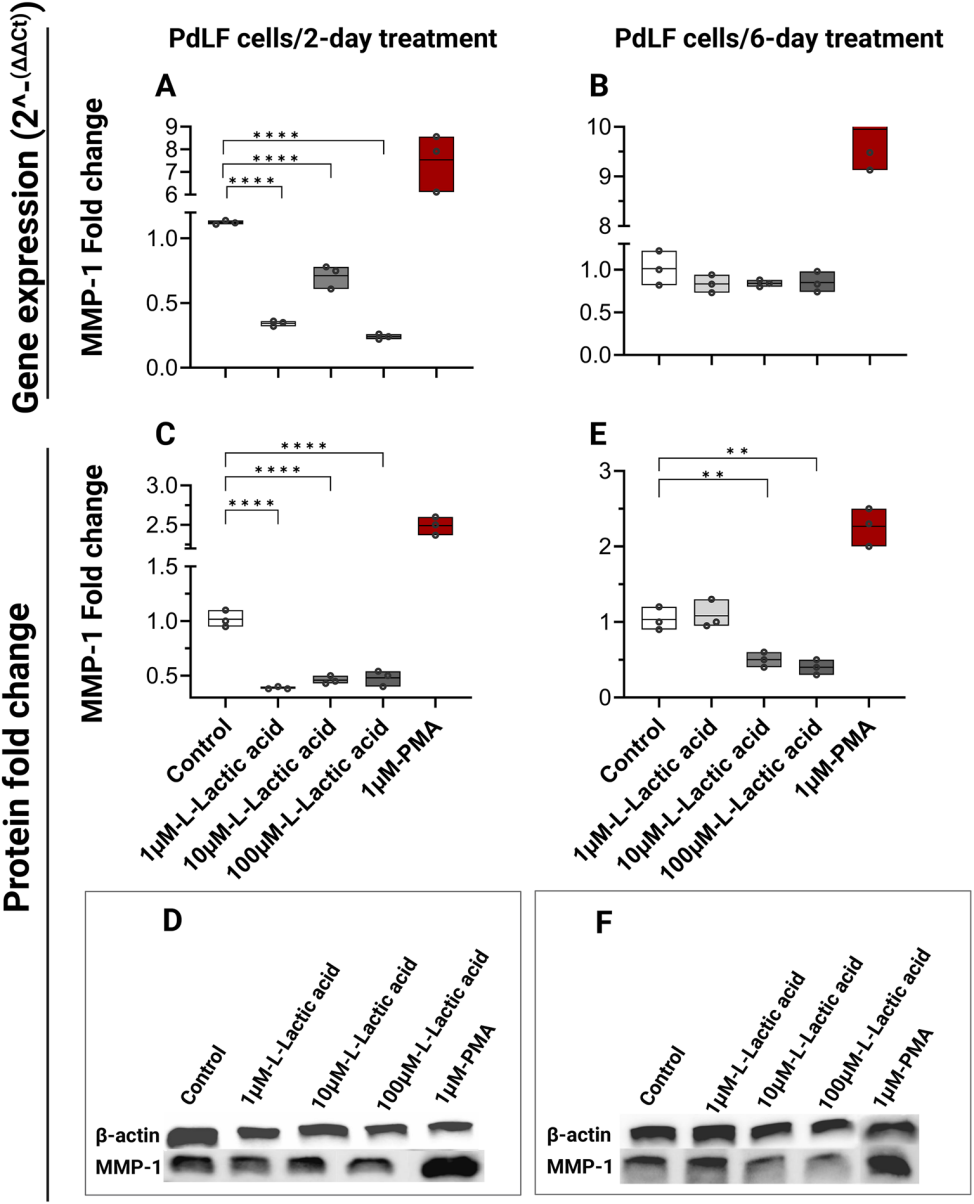
MMP-1 gene expression and protein production from PdLFs in response to L-lactic acid treatment. PdLFs were treated with 1 µM, 10 µM, and 100 µM L-lactic acid for 2 and 6 days. 1 µM phorbol 12-myristate 13-acetate (PMA) was used as a positive control for MMP-1 expression and production. The figure shows the fold change in gene expression of MMP-1 in PdLFs after (**A**) 2 days, and (**B**) 6 days of treatment. The figure shows the fold change and the protein bands of the cropped western blot of MMP-1 after (**C,D**) 2 days, and (**E,F**) 6 days of treatment. β-actin was used for western blot normalization. The cells were treated in triplicate. The data were analyzed using one-way ANOVA and Dunnett's multiple comparisons test. *P*-value < 0.05 was considered significant.

Gene expression of MMP-1 was significantly downregulated after treatment with 1 µM, 10 µM, and 100 µM (*P*˂0.0001) for 2 days (Fig. 5A), but there was no significant downregulation after 6 days (Fig. 5B). In accordance, protein abundance of MMP-1 showed significant reduction at 1 µM, 10 µM, and 100 µM after 2-day treatment (*P*˂0.0001) (Fig. 5C,D) and at 10 µM and 100 µM (*P*˂0.01) after 6-day treatment (Fig. 5E,F). The original uncropped blots are presented in the Supplementary Fig. 4.

### l-lactic acid did not induce direct M2 polarization in macrophages

Treatment of PBMCs with 1 µM, 10 µM, and 100 µM l-lactic acid caused significant polarization to classical phenotypes (CD14^high^/CD16^low^), classical/HLA-DR, classical/HLA-DR/CD86, classical/CD86, and classical/CD163 (*P*˂0.0001) (Supplementary Fig. 5A). Classical/CD206 was significantly increased at 10 µM but decreased at 100 µM l-lactic acid treatment (*P*˂0.0001) (Supplementary Fig. 5A). The non-classical phenotype (CD16^high^/CD14^low^)/HLA-DR, non-classical/HLA-DR/CD86, and non-classical/CD86 were significantly upregulated at all concentrations of l-lactic acid, while the non-classical/CD163 was significantly upregulated at 1 µM and 10 µM l-lactic acid (*P*˂0.0001) (Supplementary Fig. 5B).

### The release of cytokines from PBMCs was correlated to l-lactic acid’s effect

The release of cytokines from PBMCs following the treatment of l-lactic acid at different concentrations was tested (Supplementary Fig. 6A–L). Compared to untreated cells, l-lactic acid did not induce any significant change in the concentrations of IL-1β, and IL-6 (Supplementary Fig. 6A,B), all concentrations of l-lactic acid showed significant decrease in the release of IL-10, (Supplementary Fig. 6C), and only 10 µM l-lactic acid induced a significant reduction in IL-12p70 (Supplementary Fig. 6D). Although IL-13 was not changed (Supplementary Fig. 6E), CXCL9 and CXCL10 showed significant reduction at all concentrations of l-lactic acid (*P*˂0.0001) (Supplementary Fig. 6F,G). CCL17 did not show any significant change in the treated PBMCs (Supplementary Fig. 6H), however, both 10 µM and 100 µM l-lactic acid significantly reduced the release of CCL22, IL-23, and TNF-α (Supplementary Fig. 6I–K). Finally, IFN-γ was not changed at any concentration of l-lactic acid (Supplementary Fig. 6L).

## Discussion

### 1-Nonadecene modulates a pre-invasive microenvironment that may participate in the formation of radicular cyst and related MET phenomenon

**Figure 6:**
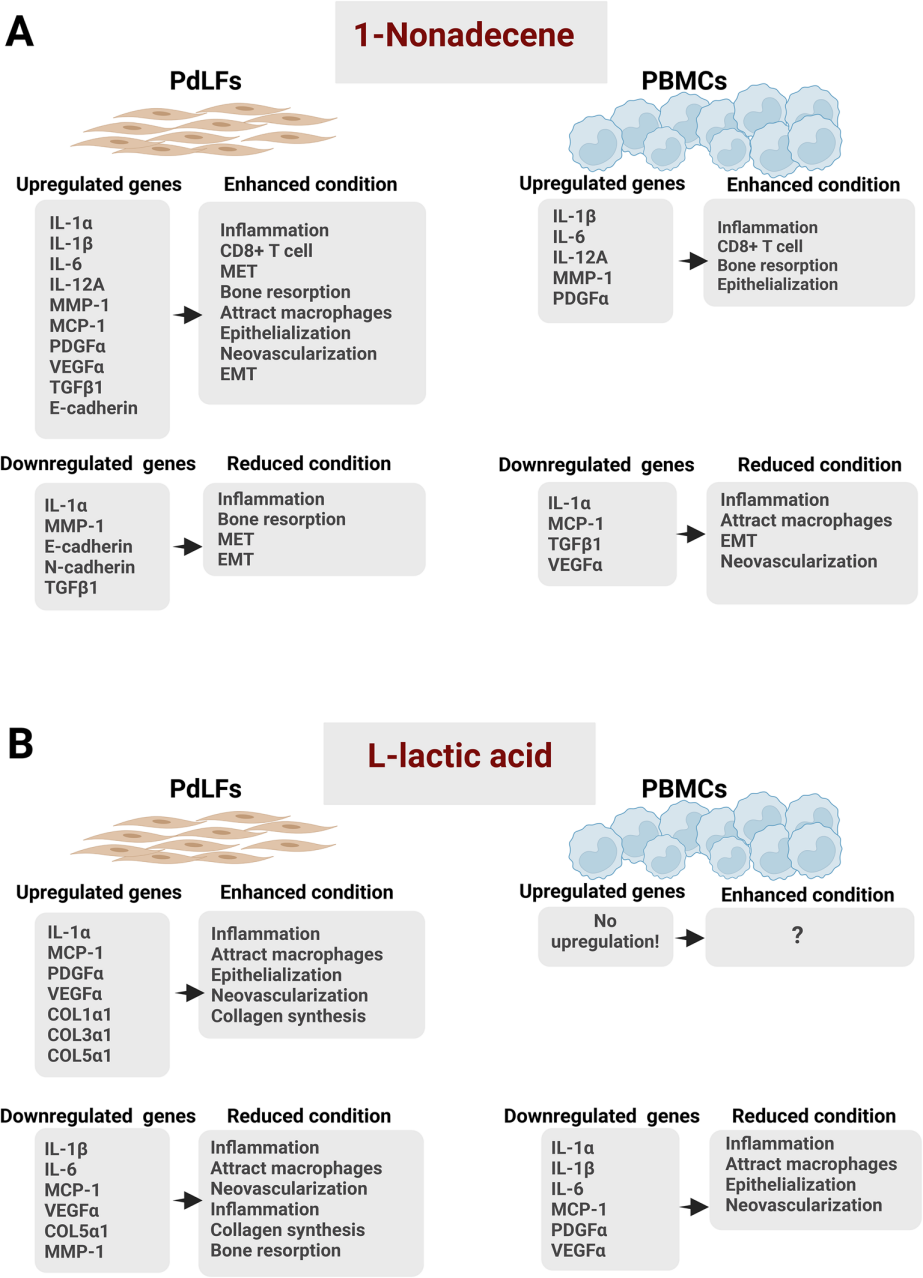
Schematic representation of the upregulation and downregulation of targeted genes in PdLFs and PBMCs. In PdLF cells, representative upregulated and downregulated genes were measured after treatment with 1-nonadecene and L-lactic acid in comparison to untreated cells for 2 and 6 days. In PBMC cells, the upregulated and downregulated genes were measured after treatment with 1-nonadecene and L-lactic acid in comparison to untreated cells for 7 days. The downstream expression of these genes are as follows: IL-1α and IL-1β enhance inflammation, MMP-1 enhances bone resorption, MCP-1 attracts macrophages, IL-12A causes clonal expansion of CD8 + T cells, TGFβ1 enhances epithelial-mesenchymal transition (EMT), upregulation in E-cadherin and/or downregulation in N-cadherin enhance MET, COLs represent collagen synthesis, and PDGFα and VEGFα increase epithelialization and neovascularization, respectively. The figure illustrates the effect on PdLF and PBMC cells in response to the treatment with (**A**) 1-Nonadecene and (**B**) L-lactic acid.

1-Nonadecene has been identified as a microbial metabolite^5,6^, however, its biological activity is still unknown. Our previous study showed that 1-nonadecene was the highest unique metabolite identified in the radicular cysts^3^. Interestingly, further investigation confirmed that the metabolite’s effect on inducing an inflammatory microenvironment is compatible with previous clinical findings^3^. IL-1β, IL-6, and IL-12A were upregulated in PdLFs and PBMCs, while IL-1α was only upregulated in PdLFs. IL-1α and IL-1β gene expressions reflect the inflammatory reaction occurring in the radicular cysts^20^. Further, IL-6 plays an important role in the pathogenesis of radicular cysts. IL-12A is important in CD8 + T cell clonal expansion and the generation of memory CD8 + T cells^21^. Autophagy pathway was the highest enriched metabolic pathway in radicular cysts^3^ and was found as a marker of cytotoxic CD8 + T cells^22^. 1-Nonadecene upregulated MMP-1 gene expression in PdLFs and PBMCs. MMP-1 contributes to bone resorption and cyst expansion. Epithelial proliferation was noticed to be high in radicular cysts^23^. For insistence, 1-nonadecene caused the upregulation of VEGFα and PDGFα. VEGFα has been reported to be an important factor in angiogenesis, cell proliferation and differentiation, and microvascular permeability. This can lead to extravasation of plasma proteins, fluid accumulation, and edema in radicular cysts^24^. PDGFα is necessary for the stabilization and maturation of the newly formed blood vessels in addition to endothelial cell differentiation^25^ (Fig. 6A).

Previous study believed that the epithelial lining of radicular cysts resulted from the stimulation of epithelial rests of Malassez in the periodontal ligaments^10^. Here, we confirmed that this pathogenesis is correlated to MET phenomenon by measuring the expression of E-cadherin and N-cadherin, consistent with previous report^26^. Epithelial cells are predominant in the lining of radicular cysts^9^ and can be derived from mesenchymal fibroblast cells^11^. Both N-cadherin and E-cadherin are affected by 1-nonadecene treatment, concluding that the same pathway is involved at both the protein and gene expression levels (Fig. 2). Previous studies showed that runt-related transcription factor (RUNX)-2 is highly expressed in radicular cyst lining^27^ and it simultaneously upregulates E-cadherin and downregulates N-cadherin, while TGFβ1 reverses this expression^28^. TGFβ1 was downregulated in PdLFs and PBMCs after treatment with 1-nonadecene (Fig. 1M,Y), which was clinically confirmed by the significantly low level in radicular cysts^29^. These findings reveal that 1-nonadecene stimulates the expression of RUNX2 that mediates the MET and at the same time downregulates TGFβ1 for further MET (Fig. 6A).

The transcriptomic results revealed a proinflammatory effect of 1-nonadecene on PBMCs (Fig. 1S–AA). Furthermore, 1-nonadecene upregulates the expression of CD14^high^/CD16^low^, HLA-DR/CD86, CD163, and CD206 in M1 macrophages, indicating a high polarization towards M1 and to be activated for phagocytosis and healing process at the same time. This result indicates the possible participation of reactive oxygen species-producing M1 in bone resorption and cystic expansion^30^. In comparison to periapical granulomas, HLA-DR was significantly upregulated in the radicular cysts^31^, while CD86 expression has not been identified previously in radicular cysts and needs further investigation. The activated pathway induced by 1-nonadecene still needs to be further investigated in the future.

Collectively, 1-nonadecene modulates a microenvironment that may be involved in the induction of the radicular cyst and the transformation of PdLFs to epithelial cells, thereby forming the cyst's lining.

### l-lactic acid modulates a pre-invasive microenvironment that may enhance the formation of periapical granuloma and related fibrosis

Although l-lactic acid was observed to cause facial granulomas after cosmetic poly-l-lactic acid injection^4^, no study investigated its pathogenic immunological role in the formation of granulomatous lesions in other body parts. In this study, we found that PdLFs and PBMCs treated with l-lactic acid exert differential anti-inflammatory and proliferative effects. In consistence, microbial lactic acid downregulates IL-1β and IL-6 in epithelial cells^32^. Although the direct effect of l-lactic acid on IL-1α in PdLFs or PBMCs has not been studied before, we observed an upregulation of IL-1α in the late treatment of PdLFs (6 days), but downregulation in PBMCs. MCP-1 showed both patterns of upregulation and downregulation at different concentrations, time points^33^, and cell types^34^. Additionally, some studies showed a proliferative effect of lactic acid through the elevated levels of VEGFα^35^ and PDGFα^36^ in macrophages, while in our study, these markers were downregulated in PBMCs. On the other hand, we observed both upregulation and downregulation of VEGFα and PDGFα in PdLFs. These results were expected since we have used different concentrations and times for the treatment (Fig. 6B).

Another promising result regarding the metabolic shift induced by l-lactic acid is the increase in collagen precipitation, which is accompanied by a simultaneous decrease in MMP-1. A high level of MMP-1 was found in aerobic glycolysis, while oxidative phosphorylation enhances collagen synthesis^37^. Hence, we concluded that l-lactic acid may induce oxidative phosphorylation in periapical granulomas represented by high collagen precipitation and low ability to degrade this excess in the collagen matrix (Fig. 6B).

The effect of l-lactic acid on PBMCs revealed that it did not induce direct M2 polarization without concomitant M1 polarization. Previously, it has been shown that lactic acid can induce M2 polarization in an environment with previous inflammation^38^. In our study, there was no previous inflammatory stimulation of PBMCs; therefore, we concluded that direct l-lactic acid treatment induces M1 polarization with concomitant activation markers, HLA-DR, CD86, and CD163 for both M1 and M2 macrophages. This means that there is an activation of both classical and non-classical macrophages, but whether this activation is sufficient to release their respective cytokines is not known. Therefore, we measured the concentration of the cytokines in PBMCs’ supernatants. The anti-inflammatory cytokines IL-10 and CCL22 were found to be downregulated in PBMCs. The anti-inflammatory effect of l-lactic acid is represented by inhibiting the release of IL-12 and TNF-α from PBMCs, consistent with other studies for this inhibition from dendritic cells and macrophages^38^. l-lactic acid inhibits the release of CXCL9 and CXCL10 in PBMCs. Ultimately, this inhibition mediates immunosuppression in periapical granulomas. l-lactic acid polarizes CD4 + T cells to Th-17 T cells^39^, but here we did not find that l-lactic acid induces the release of IL-23 from PBMCs to induce Th-17 differentiation. Overall, these results indicate that l-lactic acid alone inhibits the release of both proinflammatory and anti-inflammatory cytokines from PBMCs.

In summary, we demonstrated that a dominant unique metabolite could modulate a pre-invasive microenvironment that may participate in the initiation and pathogenesis of periapical lesions. 1-Nonadecene enhanced MET through the upregulation of E-cadherin and downregulation of both N-cadherin and TGFβ1. l-lactic acid increased the collagen synthesis and decreased its degradation by inhibiting MMP-1. Taken together, understanding the metabolomic consequences can dismantle disease’s pathogenicity, map the significance of microbial metabolites in modulation of a microenvironment, and provide a suggested therapeutic target following further clinical investigation.

## Materials and methods

### Ethical statement

PBMCs were isolated from healthy human blood samples. Ethical approval was granted by the Research Ethics Committee at the UOS for with the reference number REC-19-07-19-01 on 03/11/2019. Informed consent was obtained from all volunteers. All methods were performed in accordance with the relevant guidelines and regulations.

### Cell culture and treatment

Human PdLFs (iCell Bioscience, China) were cultured in Dulbecco's Modified Eagle medium/nutrient mixture F-12 (DMEM/F-12) (Sigma-Aldrich, Germany) supplemented with 10% fetal bovine serum (FBS) (Sigma-Aldrich, Germany) and 1.5% penicillin–streptomycin (Sigma-Aldrich, Germany). PdLFs were treated in triplicates with 1 µM, 10 µM, and 100 µM 1-nonadecene (TCI, US-Japan) and l-lactic acid^40,41^ (Sigma-Aldrich, Germany). Since 1-nonadecene has not been studied before in any experiment, we selected the same concentrations of l-lactic acid. PdLFs were incubated at 37 °C and 5% CO_2_ for 2 days and 6 days when treated with l-lactic acid^42–45^. For 1-nonadecene, the treatment regimen followed a clinical study regarding the MET phenomenon^46^ with some modifications, including the daily treatment of the PdLFs with fresh media containing 1-nonadecene. For MET induction in 1-nonadecene treatment, 2-day and 6-day treatments were used to check the irreversibility of MET induction^47^. Phorbol 12-myristate 13-acetate (PMA) (ab120297, Abcam, UK) at 1 µM^48^ was used as an inflammatory inducer^49^, a positive control for the expression and synthesis of collagen^50^, MMP-1^51^, N-cadherin^52^, and as a negative control for the expression of E-cadherin^53,54^.

Fresh PBMCs were isolated using histopaque gradient separation (Sigma-Aldrich, Germany) according to the manufacturer’s instructions and Soliman et al. 2020^55^. PBMCs were cultured in Roswell Park Memorial Institute (RPMI) 1640 medium (Sigma-Aldrich, Germany) supplemented with 10% FBS and 1.5% penicillin–streptomycin. PBMCs were treated in triplicates with 1 µM, 10 µM, and 100 µM 1-nonadecene and l-lactic acid. These concentrations were chosen to be compatible with the concentrations used in the treatment of PdLFs. For better differentiation of the PBMCs, the incubation period was extended to 7 days^56^. Lipopolysaccharide (LPS) (L2630/Sigma-Aldrich, Germany) at 100 ng/ml^57^ was used as a positive control for M1 polarization^57^ and 15 ng/ml IL-4^58^ (204-IL/R&D Systems, USA) was used as a positive control for M2 polarization^54^.

### Gene expression analysis

PdLFs, and PBMCs were used to quantify gene expression. RNAs from the lysate of cells were extracted using RNeasy Mini Kit (Qiagen, Germany), then reverse transcribed to cDNA using SuperScript™ III first-strand synthesis system (ThermoFisher Scientific, USA) according to the manufacturer’s instructions. qRT-PCR setup and cycling procedures were done as previously described^59^ using QuantStudio RT-PCR (Applied biosystems, USA). IL-1α, IL-1β^20^, IL-6, IL12A^3^, MMP-1^60^, MCP-1^61^, TGFβ1^29^, PDGF α^62^, and VEGF α^63^ are differentially expressed in radicular cysts and correlated to epithelial cells’ proliferation. IL-1α, IL-1β^64^, IL-6^3^, MCP-1^65^, PDGFα^66^, and VEGFα^3,67^ are differentially expressed in periapical granulomas. Primers of IL-1α, IL-1β, IL-6, IL-12A, MMP-1, MCP-1, TGFβ1, PDGFα, VEGFα, E-cadherin, N-cadherin, collagen (COL)1α1, COL3α1, and COL5α1 were used to study their gene expression in PdLFs and/or PBMCs. Glyceraldehyde-3-phosphate dehydrogenase (GAPDH) was used as housekeeping gene for normalization to other target genes and the relative fold change was calculated using 2^^−(ΔΔCt)^. The sequences of the employed primers are described in Supplementary Table 1.

### Flow cytometry

PdLFs were treated with 1 µm, 10 µm or 100 µm 1-nonadecene, in addition to 1 µm PMA. The cells were harvested and fixed in stain wash buffer (SWB) (1% sodium azide, 2% FBS in PBS) and the cells were then treated with rabbit anti-human E-cadherin Alexa 647 (Cat# 9835, RRID:AB_10828228, Cell Signaling Technology, USA) or rabbit anti-human N-cadherin (Cat# ab245117, RRID:AB_2910595, Abcam, USA) according to the manufacturer's instructions. Secondary antibody goat anti rabbit IgG Alexa 488 was used for N-cadherin (Cat# ab150077, RRID:AB_2630356, Abcam, USA). Corresponding isotype controls were used to compare the expression of the markers.

For PBMCs, the cells were treated with 1 µM, 10 µM or 100 µM 1-nonadecene, l-lactic acid, 100 ng/ml LPS, and 15 ng/ml IL-4. Harvested cells were treated with the anti-CD14-APC-Cy7, anti-CD16-Alexa 700, anti-HLA-DR-PE-Cy7, anti-CD86-PE, anti-CD163-PerCP-Cy5.5, and anti-CD206-APC (BD Biosciences, USA) in SWB and incubated according to the manufacturer's instructions. The stained cells were fixed in fixation and permeabilization solution (BD Biosciences, USA). PdLFs and PBMCs were then acquired in the BD FACSAria III flow cytometer (BD Biosciences, USA) using FACSDiva software with standard configuration. Compensation for PBMCs was performed by using BD CompBeads. Analysis and characterization of the markers were performed using BD FlowJo software (RRID:SCR_014422, V 10.8.0). The gating strategies was performed as previously described^68^.

### Cytokine luminex assay

Cytokine assay was performed on the supernatant of treated PBMCs with 1 µM, 10 µM or 100 µM 1-nonadecene, l-lactic acid, 100 ng/ml LPS, and 15 ng/ml IL-4. PBMCs were treated in triplicate for human cytokines detection using a magnetic bead-based multiplex assay (R&D Systems, USA) of the Luminex platform as per the manufacturer’s instructions. A total of 12 cytokines were assessed in this study including those known in M1 polarization such as IL-1β, IL-12p70, CXCL9, CXCL10, IL-23^69^, tumor necrosis factor (TNF)-α^70^, and IFN-γ^71^. IL-10, IL-13^72^, CCL17^73^, and CCL22^70^ were used for M2 polarization and IL-6^69,74^ as a pleotropic cytokine. The fluorescence of the beads was measured using a Luminex BioPlex 200 analyzer (Bio-Rad Laboratories, USA), and the data analysis was performed using BioPlex manager software (RRID:SCR_014330, BioHercules, USA).

### Collagen assay

Soluble collagen assay kit (ab242291, Abcam, UK) was used to detect the collagen formation in 1 × 10^6^ PdLFs treated with 1 µM, 10 µM, or 100 µM l-lactic acid in comparison to 1 µM PMA for 2 and 6 days. The experiment was conducted in triplicates, and the manufacturer’s instructions were followed.

### Western blot assay

To investigate the protein expression of MMP-1 in PdLFs, the cells were treated with 1 µM, 10 µM or 100 µM l-lactic acid compared to 1 µM PMA for 2 and 6 days and the western blot was performed according to Hamoudi et al.^75^. The cell pellets were lysed in RIPA buffer (ab156034, Abcam, UK) supplemented with 1:10 protease and phosphatase inhibitor cocktail-EDTA free (ab201119, Abcam, UK). The protein lysates were quantified using the Pierce BCA protein assay kit (Thermo-Scientific, USA). 10 µg Protein samples were used for MMP-1 detection using MMP-1 (54,376) rabbit monoclonal antibody and normalized with β-actin (13E5) rabbit monoclonal antibody according to the manufacturer’s instructions. The blots were visualized using the Clarity Western ECL Substrate (Bio-Rad, USA) in the ChemiDoc Touch Gel and Western Blot Imaging System (Bio-Rad, USA). Image Lab Software (V 6.1.0) was used to detect and quantify the protein bands.

### Statistical analysis

Statistical analysis was performed using GraphPad Prism software (version 9.1.0). Data were analyzed by one-way and two-way analysis of variance (ANOVA) using Dunnett's multiple comparisons tests as indicated per each graph. *P*-value < 0.05 was considered significant. * Reveals that *P*-value < 0.05, ** reveals that *P*-value < 0.01, *** reveals that *P*-value < 0.001, **** reveals that *P*-value < 0.0001.

## Supplementary Information

Supplementary Information.

## Data Availability

All data generated or analyzed during this study are included in this published article and its supplementary information files. The qRT-PCR datasets generated and/or analyzed during the current study are available in the ArrayExpress/Annotare 2.0 repository under the following accession numbers: E-MTAB-12926 for PBMCs treated with 1-nonadecene available at https://www.ebi.ac.uk/biostudies/arrayexpress/studies/E-MTAB-12926?query=E-MTAB-12926, E-MTAB-12927 for PBMCs treated with l-lactic acid available at https://www.ebi.ac.uk/biostudies/arrayexpress/studies/E-MTAB-12927?query=E-MTAB-12927, E-MTAB-12931 for PdLFs treated with 1-nonadecene available at https://www.ebi.ac.uk/biostudies/arrayexpress/studies/E-MTAB-12931?query=E-MTAB-12931, and E-MTAB-12932 for PdLFs treated with l-lactic acid available at https://www.ebi.ac.uk/biostudies/arrayexpress/studies/E-MTAB-12932?query=E-MTAB-12932.
