# Supplementary material for: Molecular pathogenicity of 1-nonadecene and l-lactic acid, unique metabolites in radicular cysts and periapical granulomas
Source: Sci Rep. 2023 Jul 3;13:10722. doi: 10.1038/s41598-023-37945-w (PMC10318002; doi:10.1038/s41598-023-37945-w)
Supplement: Supplementary file 1 — Supplementary Information. [file 41598_2023_37945_MOESM1_ESM.docx]

**Molecular pathogenicity of 1-nonadecene and L-lactic acid, unique metabolites in radicular cysts and periapical granulomas**

Alaa M. Altaie, Mohammad G. Mohammad, Mohamed I. Madkour, Mohammed Amjed AlSaegh, Manju Nidagodu Jayakumar, Aghila Rani K.G, A.R. Samsudin, Rabih Halwani, Rifat A. Hamoudi^*^, and Sameh S.M. Soliman^*^

**Supplementary information**

**Supplementary Table 1.** Primers sequences used in this study.

| Gene | Accession number | Forward primer (5′~3′) | Reverse primer (5′~3′) | Reference |
| --- | --- | --- | --- | --- |
| *IL-1α* | NM_000575.5 | CGCCAATGACTCAGAGGAAGA | AGGGCGTCATTCAGGATGAA | [^1^](#_ENREF_1) |
| *IL-1β* | NM_000576.3 | GCACGATGCACCTGTACGAT | CACCAAGCTTTTTTGCTGTGAGT | [^2^](#_ENREF_2) |
| *IL-6* | NM_000600.5 | AGACAGCCACTCACCTCTTCAG | TTCTGCCAGTGCCTCTTTGCTG | [^3^](#_ENREF_3) |
| *IL-12A* | NM_000882.4 | GCTGGCAGTTATTGATGAGC | GCATGAAGAAGTATGCAGAGC | [^4^](#_ENREF_4) |
| *MMP-1* | NM_001145938.2 | GCTGGGAGCAAACACATC | GGTGTGACATTACTCCAGAG | Current study |
| *MCP-1* | NM_002982.4 | GAATCACCAGCAGCAAGTG | GTCCATGGAATCCTGAAC | Current study |
| *TGFβ1* | NM_000660.7 | TACCTGAACCCGTGTTGCTCTC | GTTGCTGAGGTATCGCCAGGAA | [^5^](#_ENREF_5) |
| *PDGFα* | NM_001395363.1 | CGACTCCTGGAGATAGAC | GCTTCTCTTCCTCCGAATG | Current study |
| *VEGFα* | NM_001025366.3 | GCAGAATCATCACGAAGTGGTG | CATCAGGGTACTCCTGGAAGAT | [^4^](#_ENREF_4) |
| *E-cadherin* | NM_001317184.2 | ATTTTTCCCTCGACACCCGAT | TCCCAGGCGTAGACCAAGA | [^6^](#_ENREF_6) |
| *N-cadherin* | NM_001792.5 | GCGTCTGTAGAGGCTTCTGG | GCCACTTGCCACTTTTCCTG | [^7^](#_ENREF_7) |
| *COL1A1* | NM_000088.4 | CTCAAGATGTGCCACTCTGAC | CCAGTCTCCATGTTGCAG | Current study |
| *COL3A1* | NM_000090.4 | CTCAGTGGAGAACGTGGTC | CACCAGGAGATCCATCTCG | Current study |
| *COL5A1* | NM_000093.5 | CTTCCTCTACGAGGACCAC | CAGTCGAGGATCAAGGTGAC | Current study |
| *GAPDH* | NM_001256799.3 | GTCTCCTCTGACTTCAACAGCG | ACCACCCTGTTGCTGTAGCCAA | [^8^](#_ENREF_8) |

**
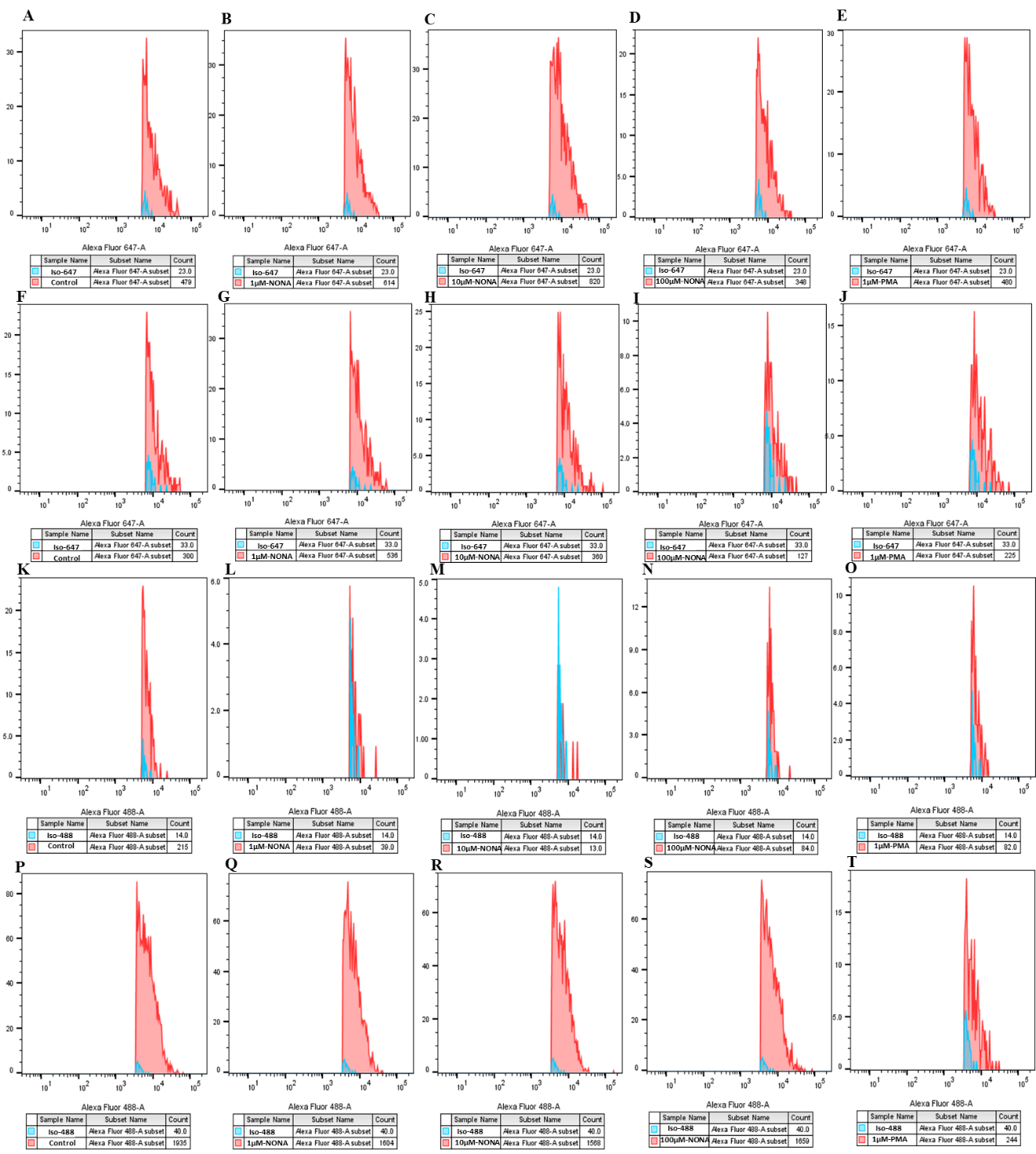
**

**Supplementary Fig. 1.** Gating strategies for PdLFs analysed in flowcytometry assay. PdLFs were treated with 1µM, 10µM, 100µM 1-nonadecene (NONA), and 1µM PMA for (**A-J)** 2 days and for (**K-T)** 6 days. Samples were compared to their isotypes **(A-J)** Alexa fluor 647 for E-cadherin antibody and (**K-T)** Alexa fluor 488 for N-cadherin. This comparison was done with **(A**, **F**, **K**, and **P)** control untreated cells, **(B**, **G**, **L**, and **Q)** 1µM 1-nonadecene, **(C**, **H**, **M**, and **R)** 10µM 1-nonadecene, **(D**, **I**, **N**, and **S)** 100µM 1-nonadecene, and **(E**, **J**, **O**, **and T)** 1µM PMA.


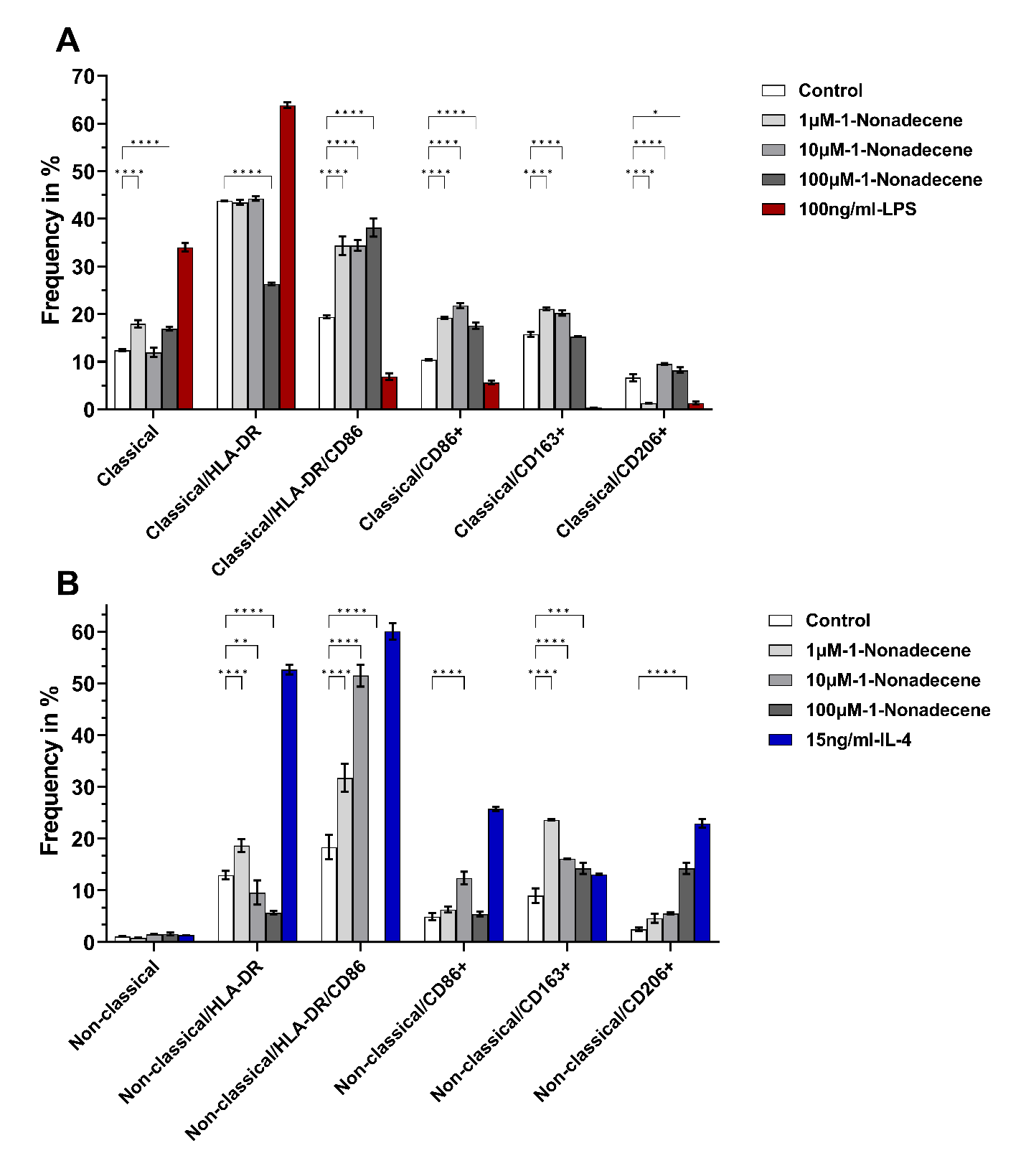


**Supplementary Fig. 2.** PBMCs polarization and activation in response to 1-nonadecene treatment. PBMCs were treated with 1µM, 10µM, and 100µM 1-nonadecene treatment for 7 days. 100ng/ml LPS and 15ng/ml IL-4 were used as positive controls for classical and non-classical polarization, respectively. **(A)** Classical polarization and activation. **(B)** Non-classical polarization and activation. All cells were treated in triplicate. The data were analyzed using two-way ANOVA and Dunnett's multiple comparisons test. *P*-value<0.05 was considered significant.


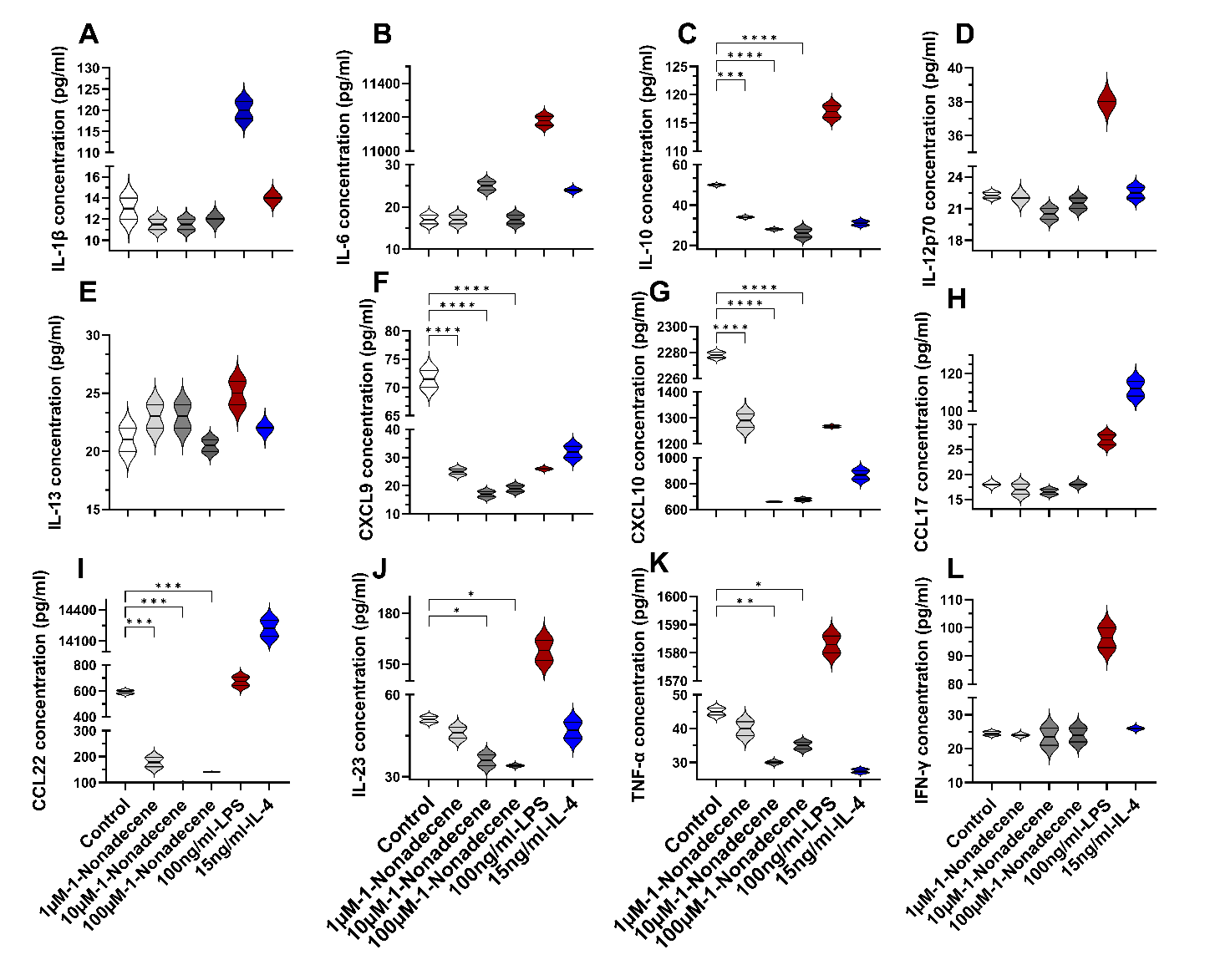


**Supplementary Fig. 3.** PBMCs’ cytokines release in response to 1-nonadecene treatment. PBMCs were treated with 1µM, 10µM, and 100µM 1-nonadecene treatment for 7 days. 100ng/ml LPS and 15ng/ml IL-4 were used as proinflammatory and anti-inflammatory controls, respectively. PBMCs’ supernatants were used to measure the proteins using Luminex assay. **(A)** IL-1β. **(B)** IL-6. **(C)** IL-10. **(D)** IL-12p70. **(E)** IL-13. **(F)** CXCL9. **(G)** CXCL10. **(H)** CCL17. **(I)** CCL22. **(J)** IL-23. **(K)** TNF-α. **(L)**. IFN-γ. All cells were treated in triplicate. The data were analyzed and one-way ANOVA using Dunnett's multiple comparisons test. *P*-value<0.05 was considered significant.


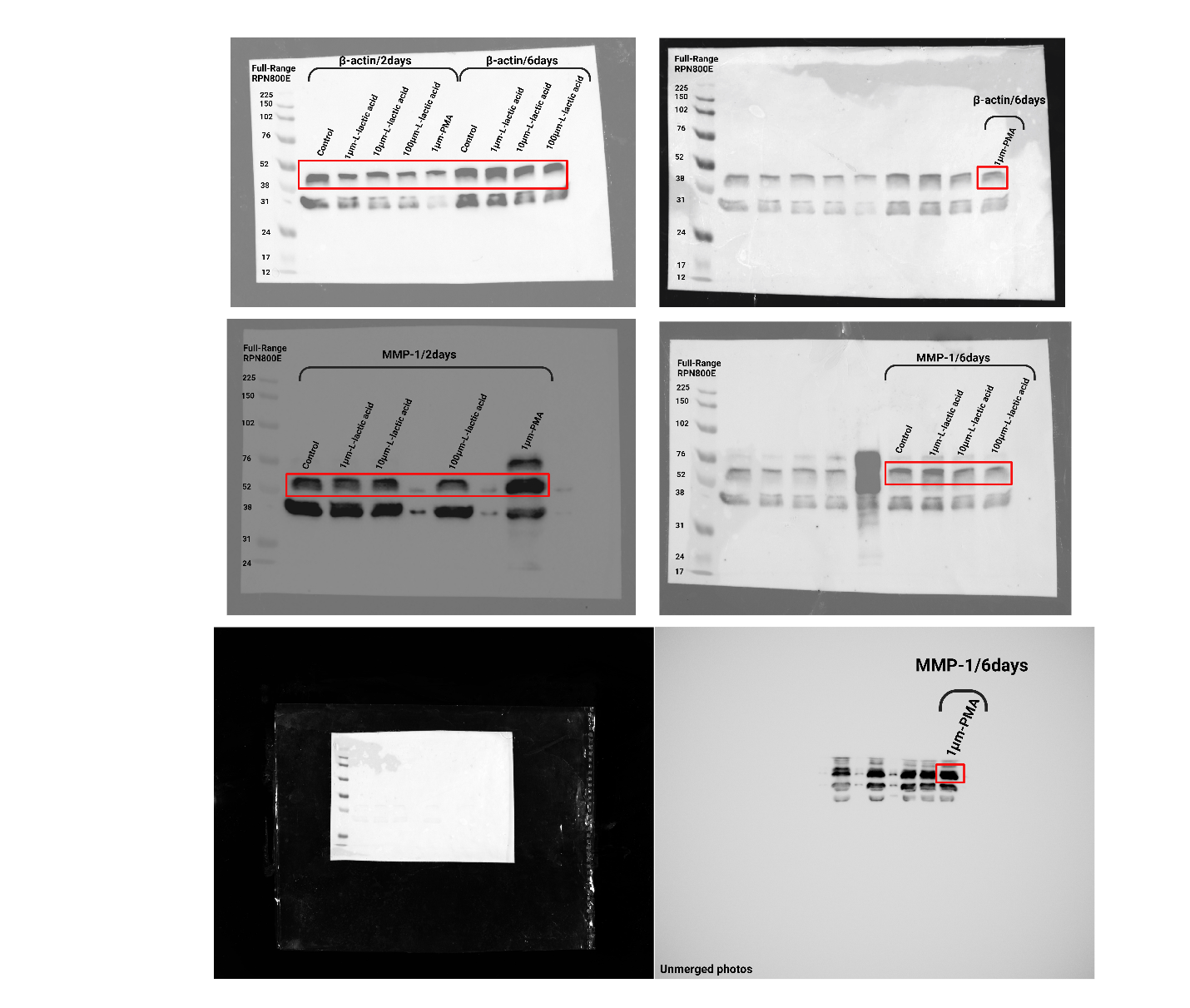


**Supplementary Fig. 4.** Original uncropped western blot of Figure 5. The figure shows the uncropped western blot of MMP-1 and β-actin proteins bands after treating PdLFs for 2 and 6 days with 1µM, 10µM, and 100µM L-lactic acid, and 1µM phorbol 12-myristate 13-acetate (PMA) in comparison to control.


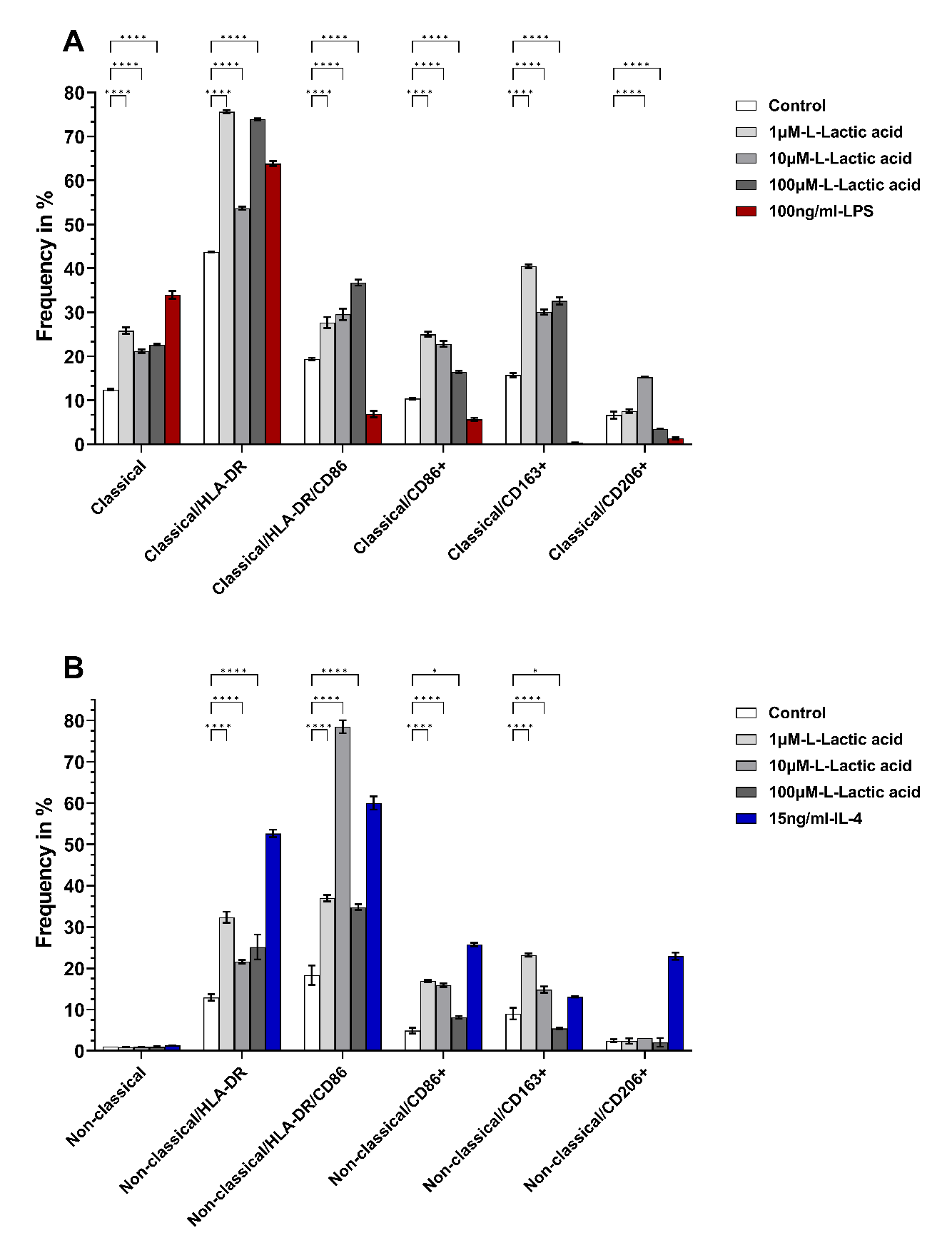


**Supplementary Fig. 5.** PBMCs polarization and activation in response to L-lactic acid treatment. PBMCs were treated with 1µM, 10µM, and 100µM L-lactic acid treatment after 7 days. 100ng/ml LPS and 15ng/ml IL-4 were used as positive controls for classical and non-classical polarization, respectively. **(A)** Classical polarization and activation. **(B)** Non-classical polarization and activation. All cells were treated in triplicate. The data were analyzed using two-way ANOVA and Dunnett's multiple comparisons test. *P*-value<0.05 was considered significant.


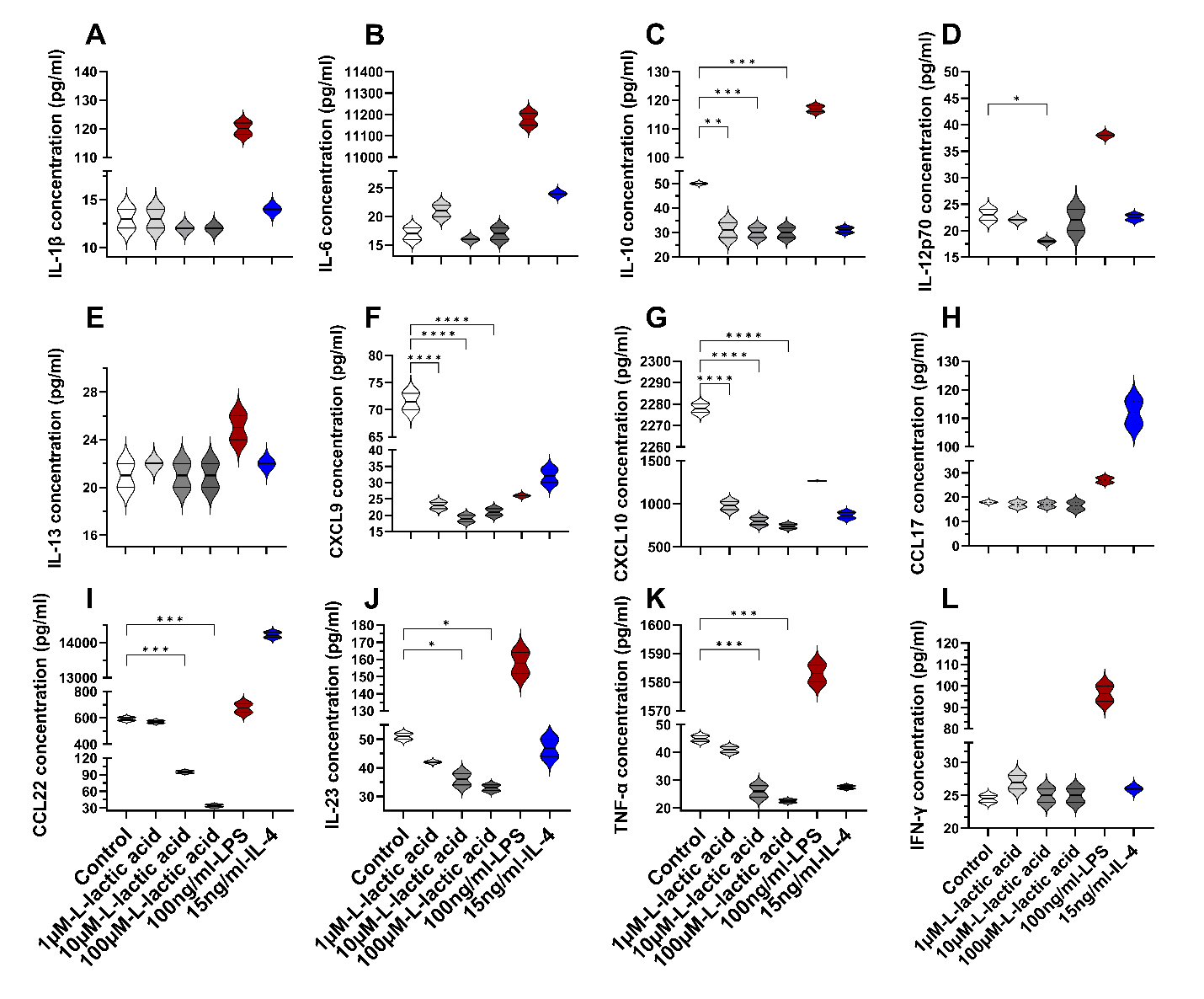


**Supplementary Fig. 6.** PBMCs’ cytokines release in response to L-lactic acid treatment. PBMCs were treated with 1µM, 10µM, and 100µM L-lactic acid for 7 days. 100ng/ml LPS and 15ng/ml IL-4 were used as proinflammatory and anti-inflammatory controls, respectively. PBMCs’ supernatants were used to measure the proteins using Luminex assay. **(A)** IL-1β. **(B)** IL-6. **(C)** IL-10. **(D)** IL-12p70. **(E)** IL-13. **(F)** CXCL9. **(G)** CXCL10. **(H)** CCL17. **(I)** CCL22. **(J)** IL-23. **(K)** TNF-α. **(L)** IFN-γ. All cells were treated in triplicate. The data were analyzed using one-way ANOVA and Dunnett's multiple comparisons test. *P*-value<0.05 was considered significant.

**References**

1 Jin, G. *et al.* An antimicrobial peptide regulates tumor-associated macrophage trafficking via the chemokine receptor CCR2, a model for tumorigenesis. *PloS One*. **5,** e10993-e10993 (2010).

2 Bhat, I. A. *et al.* Association of interleukin 1 beta (IL-1β) polymorphism with mRNA expression and risk of non small cell lung cancer. *Meta Gene.* **2,** 123-133 (2014).

3 Balint, B. *et al.* Seno-destructive smooth muscle cells in the ascending aorta of patients with bicuspid aortic valve disease. *EBioMedicine*. **43,** 54-66 (2019).

4 Altaie, A. M., Venkatachalam, T., Samaranayake, L. P., Soliman, S. S. M. & Hamoudi, R. Comparative metabolomics reveals the microenvironment of common T-helper cells and differential immune cells linked to unique periapical lesions. *Front. Immunol*. **12,** (2021).

5 Liao, G. *et al.* Human platelet lysate maintains stemness of umbilical cord-derived mesenchymal stromal cells and promote lung repair in rat bronchopulmonary dysplasia. *Front. Cell Dev. Biol*. **9,** (2021).

6 Yan, W., Chen, J., Chen, Z. & Chen, H. Deregulated miR-296/S100A4 axis promotes tumor invasion by inducing epithelial-mesenchymal transition in human ovarian cancer. *Am. J. Cancer Res.*. **6,** 260-269 (2016).

7 Park, J. W., Park, J. H. & Han, J.-W. Fermented Ginseng Extract, BST204, suppresses tumorigenesis and migration of embryonic carcinoma through inhibition of cancer stem cell properties. *Molecules*. **25,** 3128 (2020).

8 Nabokina, S. M. *et al.* Molecular identification and functional characterization of the human colonic thiamine pyrophosphate transporter. *J. Biol. Chem*. **289,** 4405-4416 (2014).
